# *Salmonella*, Food Safety and Food Handling Practices

**DOI:** 10.3390/foods10050907

**Published:** 2021-04-21

**Authors:** Olugbenga Ehuwa, Amit K. Jaiswal, Swarna Jaiswal

**Affiliations:** 1 School of Food Science and Environmental Health, College of Sciences and Health, Technological University Dublin—City Campus, Central Quad, Grangegorman, D07 EWV4 Dublin, Ireland; D19124038@mytudublin.ie (O.E.); swarna.jaiswal@TUDublin.ie (S.J.); 2 Environmental Sustainability and Health Institute (ESHI), Technological University Dublin—City Campus, Grangegorman, D07 H6K8 Dublin, Ireland

**Keywords:** food safety, food handling, food hygiene, *Salmonella*, Salmonellosis, foodborne illness

## Abstract

Salmonellosis is the second most reported gastrointestinal disorder in the EU resulting from the consumption of *Salmonella*-contaminated foods. Symptoms include gastroenteritis, abdominal cramps, bloody diarrhoea, fever, myalgia, headache, nausea and vomiting. In 2018, *Salmonella* accounted for more than half of the numbers of foodborne outbreak illnesses reported in the EU. *Salmonella* contamination is mostly associated with produce such as poultry, cattle and their feeds but other products such as dried foods, infant formula, fruit and vegetable products and pets have become important. Efforts aimed at controlling *Salmonella* are being made. For example, legislation and measures put in place reduced the number of hospitalizations between 2014 and 2015. However, the number of hospitalizations started to increase in 2016. This calls for more stringent controls at the level of government and the private sector. Food handlers of “meat processing” and “Ready to Eat” foods play a crucial role in the spread of *Salmonella*. This review presents an updated overview of the global epidemiology, the relevance of official control, the disease associated with food handlers and the importance of food safety concerning salmonellosis.

## 1. Introduction

Food poisoning due to pathogens is a major issue of public health concern worldwide with countries expending many resources to overcome it. Bacterial food infections are a source of worry for developed and developing countries. In Europe, *Salmonella* and *Campylobacter* are the most important causes of foodborne illness [1,2]. The European Centre for Disease Prevention and Control, ECDC, [3] asserts that aside from campylobacteriosis which had 246,571 reported cases, *Salmonella* is responsible for the highest number of human infections causing illnesses in 91,857 people in the EU in 2018. A foodborne outbreak is defined as an “incident during which at least two people contract the same illness from the same contaminated food or drink” [3]. There were 5146 reported foodborne outbreaks in 2018 from the EU Member States resulting in illnesses to 48,365 people. *Salmonella* alone accounted for 33% of these outbreaks. 

Salmonellosis is linked to the consumption of *Salmonella*-contaminated food products mostly from poultry, pork and egg products. Poor hand washing and contact with infected pets are some of the contamination routes [4]. When infective doses are ingested, the pathogen causes sickness by colonizing the intestinal tract. The *Salmonella* outbreak in Slovakia, Spain and Poland that resulted in 1581 cases was directly linked to infected eggs [4]. It is increasingly becoming a major concern with the global push towards ready-to-eat food products [5]. This group of products is of greater concern because of the minimal heating they are subjected to. The fact they can be consumed without high heat treatment further increases the risk. 

This review presents an updated overview of the global epidemiology, the relevance of official control, the disease association with food handlers and the importance of food safety to salmonellosis. Furthermore, numerous control measures for salmonellosis have been discussed.

## 2. Salmonella

*Salmonella* is a Gram-negative bacterium that uses flagella for movement. Salmonellosis is regarded as a foodborne infection of the gastrointestinal tract and has been reported to have high incidence rates. The causative organism can pass from the faeces of an infected person or animal to healthy ones [6]. There are more than 2500 recognized serotypes [7].

*Salmonella* is known to survive for extended periods in low moisture food products [8]. Table 1 shows how long different serotypes survive in dry products. Its ability to survive in low moisture environments is a problem with spices and herbs that are used globally because if contaminated, these organisms survive for extended periods. Worldwide trade of spices and herbs means these organisms could travel and break geographical barriers [9].

### 2.1. Occurrence of Salmonella

Salmonellae live in the gastrointestinal tracts of domestic and wild animals [18]. A study by Munck et al. [4] identified nine potential sources of *Salmonella*: avian, bio solids-soil-compost, companion animals, equine, poultry, porcine, reptile, ruminant, and wildlife. Wild birds have been known to be a reservoir of these bacteria. The organism resides in the intestines of infected birds and may not cause obvious clinical symptoms except intermittent fevers. Migratory birds are a particular concern. For example, there are several points in the Ukraine where these migratory birds’ nest on their journeys between Europe to Africa and Asia [19]. These areas are considered hot spots for *Salmonella* from where the pathogen is distributed to different parts of the world.

Domestic animals are also *Salmonella* reservoirs. In 2019, it was estimated that about 12 million people, that is 40% of the households, in the UK owned pets. Dogs and cats are top on the list but exotic pets such as reptiles, birds, etc. are also kept more frequently [20]. As early as the 1940s, it was proven that humans can get *Salmonella* from reptiles [21]. Bjelland et al. [22] found that 43% of Norwegian reptiles shed *Salmonella*. The Centre for Food Security and Public Health [23] indicated that 93,000 human cases resulted from human association with reptiles. Table 2 gives an overview of salmonellosis cases associated with pets and domesticated animals. Salmonellosis is chiefly a foodborne infection but 7% of human salmonellosis is related to reptiles [23]. These reptiles carry the bacteria in their intestinal tract and shed them through their faeces. This is especially a problem when children are involved with these pets as children belong to a high-risk group. Finlay et al. [21] indicated that *Salmonella* cannot be eliminated from reptiles with the use of antibiotics, as a treatment only increase their antibiotic resistance. Humans, especially infected food handlers, and contaminated environments are also major reservoirs of *Salmonella* [24].

### 2.2. Epidemiology and Pathogenicity

The severity of *Salmonella* infections is dependent on the specific strain responsible for the infection and on the health status of the host. Children below the age of 5, the elderly and immunocompromised adults represent a specific group that is more susceptible to salmonellosis [32]. 

Salmonellosis is often characterized by stomach flu (gastroenteritis). This illness is accompanied by nausea, vomiting, abdominal cramps and bloody diarrhoea. It is also associated with headache, feverish conditions and myalgia. The continuous loss of body fluids may result in dehydration especially for infants and the elderly [23]. Salmonellosis is a self-limiting illness that ceases in a week, but deaths have been recorded especially in vulnerable population groups such as very young, elderly and immunocompromised persons [32]. Kurtz, Goggins and McLachlan [33] assert that in cases where salmonellosis becomes systemic, enteric fevers can arise after gastroenteritis and enterocolitis have waned. Enteric fever is a common symptom when *S.* Typhi is the causative organism. These cases are characterized by fever, anorexia, headache, lethargy, myalgia, constipation, and other non-specific symptoms. When resulting in septicemia or meningitis, the disease can be fatal.

Reactive arthritis (ReA) or Reiter’s syndrome is a reactive inflammation of the joints that occurs after a gastrointestinal or genitourinary infection. However, its pathogenesis is currently not fully understood [34]. It affects adults between the ages of 20–40 and symptoms may include: painful joint inflammations, eye inflammation, discomfort in urination, swollen toes and fingers, lower back pain, rash on soles and palms, etc. ReA occurs due to *Salmonella* infection in 12 cases per 1000 globally [35]. In both the USA and Europe, ReA has followed salmonellosis in about 15–17% of self-reported patients [36]. There is no agreement on the role of genetics and the risk of having this disease. However, some studies have shown a correlation between the possession of the HLA-B27 surface antigens and the severity of the disease [32].

### 2.3. Food Products Associated with Salmonella

*Salmonella* Agona is a less known *Salmonella* serovar. Between the years 2007–2016, it was responsible for 13 outbreaks resulting in 636 illnesses that required hospitalization in the EU. Nine of these outbreaks were due to the consumption of contaminated foods (Table 3). Chicken was responsible for two outbreaks in 2013, red meat for one outbreak in 2014, pork for one outbreak in 2012, unspecified poultry meat for an outbreak in 2007, mixed foods and bakery products were both vehicles for different outbreaks in 2017 [37].

In accordance with EU Zoonosis Directive 2003/99/EC, Member States are required to report sources and trends of zoonosis, zoonotic agents and foodborne outbreaks [50]. In 2016, *S.* Agona were isolated from 25 units of foods in 4 Member States and a non-Member State. Approximately 68% of these samples were from meat from poultry. Other isolates were from beef (3), pork (1), cheese from unpasteurized milk (1) and dried seeds (1) [50]. In the same year, 242 units of animals tested positive for *S.* Agona from chicken (209) and turkey (25). These were reported by 11 Member States and two non-Member States. Between the years 2004 and 2015, 608 units tested positive for *S.* Agona in different animal feeds. A majority of them were related to oil seeds or fruit origin (243), then those feeds sourced from land animals (64), another 64 came from unspecified feed sources, feeds from marine animals (43), pet foods (30) while feed for poultry accounted for 28 [37]. However, *S.* Agona occurs less in eggs and its products, fish and its products and fruits and vegetables. There was no report of it being present in “foodstuffs intended for special nutritional uses” and “infant formula” [37]. In the United States, the two most common strains remain *Salmonella* Typhimurium and *Salmonella* Enteritidis [51] but according to outbreaks reported by the CDC in 2019, other strains have been responsible for several foodborne illnesses, leading to hospitalizations and death as reported on (Table 3).

### 2.4. Salmonella and Vegetable Produce

Traditionally, plants are not recognized as hosts for human pathogens such as *Salmonella* but in the last few decades, the niches for these organisms have changed [52]. *Salmonella* produces periplasmic enzymes with the ability to break plant surface barriers. However, the penetration of these enzymes into plant systems is dependent on pectin and polygalacturonate processing (level of ripening) and physiological wounds [21,53].

Members of the Enterobacteriaceae family are capable of penetrating the stomata of plant leaves [54], hydratodes [55] and roots [56]. Plants contaminated pre- or post-harvest do not exhibit signs of spoilage [57] while the organisms contaminate the produce whether pre-harvest or post-harvest [58].

On the farm, produce is exposed to *Salmonella* by contact with wildlife, contaminated irrigation water, untreated manure [55,59,60,61,62,63]. Poor hygiene by fieldworkers, use of mobile toilets and hand-washing stations increase the risk of pathogen dissemination at pre-harvest [64] and during harvest [65]. After harvest, contamination of produce is mainly due to poor hygienic practices [63,66].

In the United States, food poisoning outbreaks from raw eggs and seafood is on a decline while outbreaks due to fruits and vegetables keep increasing [15,67], even though field surveys carried out in the United States indicated that *Salmonella* contamination is low during pre-harvest production. Fruits and vegetables have been associated with 130 outbreaks since 1996 [15,42,67,68]. Bennett et al. [69] noted that tomatoes specifically were implicated in 15 multi-state outbreaks of salmonellosis between 1990 and 2010. Traceback analysis suggested that contamination happened during the production or processing stages.

Devleesschauwer et al. [70] noted that although salmonellosis outbreaks due to fruits and vegetables have been well documented, their occurrence, however, remains sporadic. Moreover, Devleesschauwer et al. [70] also stated that for outbreaks involving fruits and vegetables to occur, a multitude of factors must come together. These factors include the presence of vectors, level of crop maturity, physiological defects, presence of native biota that may inhibit or promote human pathogens, type of irrigation practised, etc. The role of environmental conditions and farm practices is also essential in determining the factors that make plants susceptible to *Salmonella* proliferation both pre and post-harvest. The study carried out by Devleesschauwer et al. [70] confirmed that harvesting tomatoes when still green significantly reduces *Salmonella* infestation, as does harvesting after a period of high humidity. Pre-harvest application of copper, iron, potassium, nitrogen or foliar sprays did not affect post-harvest contamination.

## 3. Global Burden of Salmonellosis

Stanaway et al. [71], while reporting on the global burden of non-typhoidal *Salmonella* invasive disease, asserted that non-typhoidal *Salmonella* remains a major cause of disease and death worldwide. Malnourished young children, the elderly, immunocompromised adults (such as HIV patients), sufferers of acute malaria and those with pre-existing debilitating sickness have greater risks. This infection can attack healthy hosts and in addition to diarrhoea, causes bacteraemia, meningitis and infections in the tonsils. In 2017, *Salmonella* enterocolitis caused 95.1 million disease conditions, 3.1 million disability-adjusted life-years and 50,771 fatalities according to The Global Burden of Diseases, Injuries, and Risk Factors Study (GBD) [71]. The Foodborne Disease Burden Epidemiology Reference Group (FERG) of the WHO in 2010 reported that *Salmonella* was responsible for a total of 180M illnesses and 298,496 deaths (Table 4).

Food illnesses from invasive non-typhoidal *S. enterica* presented the highest disease burden. This is due to the pervasive nature of this organism, the acute diarrhoea it causes and frequent infection of children [74]. Kirk et al. [73] evaluated the health impact of all the serotypes of *Salmonella* and concluded that it presents the greatest foodborne burden. Combining data associated with *S. enterica* from both the invasive Non- Typhoidal *Salmonella* (iNTS), *Salmonella* Typhi and *Salmonella* Paratyphi A and diarrheal infections, a total of 8.76 million Disability-Adjusted Life Year (DALY) from all transmission sources and 6.43 million attributed to infected foods.

In France, between 2008 and 2013, disease pathogens caused between 1.28–2.23 million illnesses, 16,500–20,800 hospitalizations, and 250 deaths. *Campylobacter* spp., non-typhoidal *Salmonella* spp., and norovirus were responsible for >70% of all foodborne pathogen-associated illnesses and hospitalizations while non-typhoidal *Salmonella* spp. and *Listeria monocytogenes* were the main causes of foodborne pathogen–associated deaths. *Salmonella* spp. ranked third as the cause of foodborne illnesses (12%), second as a cause for hospitalization (24%), and first as a cause of death (27%) [75]. Furthermore, Simpson et al. [24] stated that salmonellosis is the second main cause of gastroenteritis in Australia and the most common cause of food-related deaths in the world.

In the EU, there are more than 91,000 reported *Salmonella* infections each year [76]. In 2016, there were 94,530 human cases of salmonellosis reported in the EU with *S.* Enteritidis accounting for 59% of all cases [50]. There was an increase of 11.5% in the trend of reported food outbreaks compared with that of 2015 and *S.* Enteriditis was responsible for one in six outbreaks in 2016. *Salmonella* was responsible for the highest health burden with 1766 hospitalizations (45.6%) and 50% of all deaths in outbreak cases [50]. In Australia, gastroenteritis was responsible for about $811 million annually in costs associated with treatments, deaths, loss of productive hours and government surveillance [24]. 

From 2009 to 2015, there was a drastic increase in hospitalizations due to salmonellosis among the EU/EEA Member States. Concerted efforts by the European Commission and stakeholders tried to level case numbers in 2015 at 12,510 hospitalizations. However, recent data show the trend is rising again with 16,816 recorded hospitalizations in 2018. The USDA ERS [77] estimated the economic cost of *Salmonella* (non-typhoidal) as $3.66B for 2014 to account for lost wages, medical costs, premature deaths, number of cases and productivity losses. In the EU, these costs are estimated to exceed €3 billion a year [3]. Other studies as shown in (Table 5) recorded the cost of illness caused by salmonellosis.

## 4. Control of Salmonellosis

The coordinated *Salmonella* control programs implemented by the EU are one of the most celebrated milestones for the fight against zoonotic diseases. Before 2004, there were over 200,000 reported human salmonellosis cases in 15 EU Member States but control programs put in place reduced this number to 90,000 cases annually in the whole 28 Member States [83]. This led to a reduction by half of the usual cases between 2005 and 2009. The amended EU Regulation 2073/2005 requires the absence of *Salmonella* in 25 g of pooled neck skin samples for broiler carcasses, turkey carcasses and most food types.

However, as evidenced by the Eurobarometer, Europeans are increasingly worried about food safety due to contaminations from pathogenic bacteria. The rising trend of reported cases makes activities aimed at increasing consumer awareness of these foodborne illnesses a requisite [3]. The European Union established an integrated approach to control *Salmonella* in the food chain. This approach involved players at the top government level of the EU Member States, the European Commission, the European Parliament, EFSA and ECDC [76]. The EU took a drastic step to curtail the spread of *Salmonella* by applying extended control programs and legislation that cover the routes of *Salmonella* exposure (Table 6). Under this regulation, an absence of *Salmonella* is required in ready-to-eat foods. Industrially, proof of its absence is a part of buying specifications for raw and finished products. Its absence is taken as evidence of microbiological examination done to support both HACCP control and due diligence. A microbiological criterion for *Salmonella* has been written into law for diverse foods such as poultry products, molluscs, dairy, meat and meat products, ready-to-eat foods, etc. [84].

Regulation (EC) No 2160/2003 sets a Union target for each Member State to reduce *Salmonella* in their poultry flocks from 10 to 40% based on their number in the previous year. Every country must achieve at least a 2% reduction annually. However, Regulation (EC) 270 No 517/2011 (Table 6) as amended sets a Union target of 1% or less for *Gallus gallus* breeding flocks positive for *Salmonella enteritidis*, *Salmonella infantis*, *Salmonella hadar*, *Salmonella typhimurium*, monophasic *Salmonella typhimurium* with the antigenic formula 1,4, [5],12:i:-, and *Salmonella* Virchow. Regulation 517/2011 requires sampling to be at least once every 16 weeks compared to 200/2010 which required once every 15 weeks. Commission Regulation (EU) No 1190/2012 (Table 6) which repealed 584/2008 requires that the maximum percentage of *Salmonella* Enteritidis and *Salmonella* Typhimurium should be less than or equal to 1% in both breeding and fattening turkeys. 

Curtailing the spread of *Salmonella* involves controls that start from poultry production on the farm until products get to the table of consumers. These controls have to be a farm to fork systematic set of processes [85]. The WHO in 2018 gave recommendations for control of *Salmonella* that cover the whole food chain. These efforts are aimed at strengthening food safety standards that enhance *Salmonella* surveillance efforts, educating consumers and training food handlers on best practices in preventing *Salmonella* and other foodborne diseases (Table 7). It further stressed the importance of national and regional surveillance networks in identifying and monitoring this disease to forestall its detrimental activities and halt its spread. The contact points between children and domesticated animals such as cats, dogs and pet reptiles are mentioned as requiring supervision. The WHO works in improving the effectiveness of national and regional laboratories in tackling salmonellosis. 

### 4.1. Food Hygiene Practices

Food hygiene refers to the encompassing conditions and measures that prevent food contamination from production to consumption. Poor hygiene practices along the food chain from slaughtering or harvesting, processing, storage, distribution, transportation to preparation can expose the consumer to foodborne infections that may be fatal [86]. Proper food hygiene practices centre on cleanliness, separating raw meat from other raw/cooked foods, cooking at correct temperatures and chilling (storing) foods before and after cooking [87]. The USFDA [39] reported that poor hygiene during food handling can lead to the spread of *Salmonella* in foods.

Numerous foodborne outbreaks are associated with restaurants [88]. According to CDC estimates, 59% of these outbreaks in the United States happened in the foodservice industry [89]. The CDC estimates that 48 million people suffer from food-related illness, 128,000 are hospitalized and about 3000 subsequently die each year [48]. About 75% of these cases are caused by poor food handling practices in restaurants [90,91].

The catering industry is expanding massively; from 2010 it had increased by 26.5% and this trend is not abating [92]. In 2017 alone, the industry had a revenue of USD800 billion [93]. With this level of growth due to changing societal eating habits, there arises a higher chance for outbreaks of foodborne disease. Food handlers have access to food products when they are unwrapped, the equipment used in making them and places where these unwrapped products are stored or displayed, and therefore can be potential sources of contamination. Poor handling practice at this level is a high-risk factor for foodborne outbreaks. It is therefore very important that workers have adequate food safety training to sustain the industry [94]. 

### 4.2. Food Handler Effects

The Codex Alimentarius defines a food handler as “any person who directly handles packaged or unpackaged food, food equipment and utensils, or food contact surfaces and is therefore expected to comply with food hygiene requirements” [95]. Food handlers play a major role in food production and serving. They are responsible for preparing the food and this means they have more direct contact with food systems and can invariably be agents of contamination. The chance for contamination largely depends on how healthy the food handlers are, their personal hygiene, knowledge and application of food hygiene rules [96]. Solomon et al. [97] reported on a study carried out involving 387 food handlers in a meal-serving facility. A total of 159 (41%) of the food handlers had one or more intestinal parasites and 35 *Salmonella* species were isolated from them. Another study was done in Arba Minch University students’ cafeteria in Ethiopia involving 345 participants. Stool cultures revealed that 6.9% were positive for *Salmonella* and 3% for *Shigella* [96]. The prevalence of salmonellosis amongst people and food handlers, in this case, increases the risk of food contamination by physical contact (i.e., touching the food with unwashed hands). A food handler can directly cross-contaminate food during preparation by allowing raw foods to come in contact with cooked or ready-to-eat foods or allowing blood or juices to flow from raw to the cooked foods [95]. FSAI further stressed that handlers can indirectly contaminate foods by touching cooked foods after preparing raw foods without prior washing of hands, using the same equipment and utensils meant for raw foods for cooked foods, displaying cooked foods in places meant for raw foods or by poor personal hygiene.

#### Hygienic Meat Handling Practices

*Salmonella* has been isolated from meat products more than any other foodstuff. Poultry and its products present the highest statistics on salmonellosis. Adequate meat handling practices start from the farm where these animals are raised. EC 853/2004 prohibits the transport of animals suspected to be sick, which come from herds known to be diseased, to the slaughterhouse without the permission of the competent authority. It also gives specific requirements for slaughterhouses to combat the spread of *Salmonella*. These include having hygienic and sufficient lairage facilities, lock rooms for diseased or suspected animals, separate rooms for evisceration and cutting, etc. The regulation aims at preventing contamination of meat, ensuring disinfectants are present, focuses a lot on slaughter hygiene, and mandates conditions in which the meat must be in during storage and transport [98]. The Hygiene rating of slaughterhouses is highly dependent on technical issues such as slaughter line speed, efficient work routines and the number of carcasses each operator has to deal with. Inadequacies in these factors raise the risks of food infections (Table 8). 

Despite the stringent controls used on farms and slaughterhouses, *Salmonella* is still present in the meat. The handling processes are not aimed at sterilizing the meat but instead at slowing down their activities. The moment these products are exposed to favourable conditions, the bacteria start to grow and multiply to dangerous levels. Hence, hygienic meat handling practices are crucial both domestically and in catering services. The proper handling of meat starts from purchasing raw meats from reputable vendors. If it is pre-packed, then the use-by dates must always be checked. 

Raw meat should be kept in separate bags apart from ready-to-eat foods to avoid cross-contamination. Storing of meat is a crucial step. Raw meat/poultry should be stored in sealed bags at the bottom of the fridge as early as possible [58]. This limits the time for *Salmonella* to grow and avoids the dripping of fluids to other foods. Freezing meats before the use-by dates halt the growth of bacteria. Defrosting can be done in a tray at the bottom of the fridge. It is recommended to defrost 2.5 kg/5 lbs of meat or chicken for 24 h. However, when defrosting is done in a microwave, it should be consumed right away [106]. Hands should be washed before and after handling raw meat. All meat types need to be properly cooked before consumption to avoid the intake of bacteria. For whole chicken, cooking should be at 180 °C for 20 min. The same weight for pork and rolled meats should be cooked at the same temperature but for 35 min. Verifying all parts of the meat have received adequate heating is essential. Cutting into the thickest part of the meat to see if the juice runs clear indicates adequate cooking ensuring no part is pink [106]. A thermometer or probe should be used domestically and in catering services for checking temperatures in different parts of food. Areas where meat is handled, and utensils should be colour coded.

### 4.3. Ready-to-Eat (RTE) Foods and Processed Foods with Needed Control

Processed food is defined as any food that has changed in its preparation. This alteration can be freezing, canning, heating, baking, etc. [107]. *Salmonella* has been isolated from processed foods such as nut butter, frozen pot pies, chicken nuggets, and stuffed chicken entrees [25]. Huang and Hwang [108] defined RTE foods “as a group of food products that are pre-cleaned, precooked, mostly packaged and ready for consumption without prior preparation or cooking”. The fact that RTE foods need no further heating step means the consumers have a heavy reliance on the control programs put in place by processors. RTE foods have a shorter shelf life compared to other processed foods. The shelf life is usually a maximum of three weeks after manufacture because they have not been subjected to lethal temperatures to conserve organoleptic properties. These foods depend on hurdle preservative steps such as acidic environment, packaging used, isotonic medium, refrigeration, etc. RTE foods have been linked to several salmonellosis outbreaks such as *Salmonella* Coelin in ready-to-eat salad mix [109], *Salmonella enterica* in chill ready-to-eat poultry meat products [110]. Due to the nature of RTE foods, the risk for contamination and cross-contamination leading to illness is quite high. Finished process testing is only valid for the verification process because the results could be coming in too late [9]. Moreover, the fact that a few samples taken from a batch of products pass microbiological criteria does not guarantee that all products are safe especially when heterogeneous and local contamination may occur [111]. However, food safety management programs based on prerequisite programs and HACCP covering all stages of production will ensure hygiene and microbiological criteria is met. There is a necessity for all food handlers to be trained and retrained periodically on food safety especially when dealing with RTE foods to improve knowledge of food handling and food poisoning (Table 9).

### 4.4. Knowledge vs. Behavioural Training Models

Well-trained food handlers with adequate knowledge of food safety can reduce the risk of food hazards [91]. The fact that many restaurants use different means of ensuring food safety, but outbreaks still occur frequently and are related to poor handlings, raises the question of the efficacy of such training [92]. It is often believed that increased knowledge would directly translate to best practices, but this is not always the case [88]. Training is usually focused on passing information, assessment, and certification. All these are done in a brief period without the opportunity to see it work in real practice and assess if it is translated into behaviour [92]. Yu et al. [91] note that translating knowledge to behaviour is not an easy task just as it was shown that knowledge of proper food handling and behaviour are different things [115].

McFarland et al., [92] reviewed six studies as reported in (Table 3). Results from five of the studies indicated that an increase in the knowledge of an employee on food safety does not necessarily transfer into proper food safety behaviour. Yu et al. [91] showed that knowledge-based training is good, but behaviour training is better. The best results come from a combination of both methods. Knowledge-based training influenced behaviour in some ways, but this effect did not last if used alone. It failed during peak periods in the restaurant. Participants in the behaviour-based training still carried on good practices after the training for longer periods. Husain et al. [112] focused their study on three factors that can influence behaviour: attitude, normative beliefs, and perceived behavioural control. This study centred on food handler having a clear understanding of the importance of food safety in preventing foodborne illness. If they do not understand why they do what they do, then the behaviour would not change. Results showed that there was an improvement in personal hygiene and safe preparation of food for 12 weeks but did not translate to technical procedures such as time-temperature abuse, proper sanitation, etc. [92]. It is also very important to tailor training based on the role the employee takes and their background. The language is spoken and the level of education becomes very important. Type of training material is also important such as videos instead of text, pictures instead of just words and other languages instead of English [113]. 

## 5. Future Perspective and Conclusions

Efforts to control salmonellosis should involve both the public and private sectors. Government regulations and stricter measures being put in place can provide a framework that guides both domestic production and international importation requirements. However, this has to be infused into periodic training for food handlers. Industrially, stricter control systems need to be put in place. There should be more focus on production and process controls than on testing finished products. Consumers need to be educated both formally and informally on the basic steps of food safety. There is a need for studies that identify the most suitable means of communicating scientific information and raising awareness on salmonellosis to all strata of the population. 

## Figures and Tables

**Table 1:** *Salmonella* survival times in low water activity environments.

Food	*Salmonella* Serotypes	Survival Times	Reference
Dried milk products	*S*. Infantis, *S.* Typhimurium, *S.* Eastbourne	≤10 months	[10]
Desiccated plastic surface Pasta	*S.* Typhimurium *SL 1344*, *S.* Infantis, *S.* Typhimurium, *S.* Eastbourne	<100 weeks	[11]
		≤12 months	[12]
Milk chocolate	*S.* Infantis, *S.* Typhimurium, *S.* Eastbourne	>9 months at 20 °C	[13]
Bitter chocolate	*S.* Eastbourne	≤9 months at 20 °C	[13]
Halva	*S.* Enteritidis	>8 months at refrigeration temp	[14]
Peanut butter	*S.* Agona, *S.* Enteritidis, *S.* Michigan, *S.* Montevideo, *S.* Typhimurium	≤24 weeks at 5 °C ≤6 weeks at 21 °C	[15,16]
Paprika powder	multiple serotypes	>8 months	[17]

**Table 2:** *Salmonella* outbreaks involving pets/pet foods.

*Salmonella* Strains	Pet/Pet Food Product	Cases	Locations Affected	References
*S.* Typhimurium	Small Pet Turtles	34 reported cases and 11 Hospitalizations	9	[25]
*S.* Oranienburg	Small Pet Turtles	26 reported cases and 8 Hospitalizations	14	[26]
*S.* Cerro *S.* Derby *S.* London *S.* Infantis *S.* Newport *S.* Rissen	Pig Ear Pet Treats	154 reported cases and 35 hospitalizations	35	[27]
*Salmonella* spp.	Backyard Poultry	1134 reported cases, 219 hospitalizations and 2 deaths	49	[28]
*Salmonella* spp.	Poultry in Backyard Flocks	1120 reported cases, 249 hospitalizations and 1 death	48	[29]
*S.* Reading	Paws Ground Turkey Food for Pets	90 reported cases	26	[30]
*Salmonella* spp.	Reptiles	449 hospitalizations	Ireland	[31]

**Table 3:** Food products involved in *Salmonella* outbreaks in Europe and United States.

*Salmonella* Strain	Food Product	Cases	Locations Affected	References
*S.* Javiana	Pre-cut fruits	165 reported cases and 73 hospitalizations	14	[25]
*S.* Newport	Red Onions	640 reported cases and 85 hospitalizations	43	[38]
*S.* Javiana	Fruit Mix	165 reported cases and 73 hospitalizations	14	[39]
*S.* Uganda	Cavi Brand Whole, Fresh Papayas	81 reported cases and 27 hospitalizations	9	[40]
*S.* Newport	Frozen Raw Tuna	15 reported cases and 2 hospitalizations	8	[41]
*S.* Carrau	Pre-Cut Melons	137 reported cases and 38 hospitalizations	10	[42]
*S.* Uganda	Fresh Papayas	81 reported cases and 27 hospitalizations	9	[43]
*S.* Dublin	Reblochon (bovine raw-milk cheese)	83 reported cases and 41 hospitalizations and 10^b^ deaths	France	[44]
*S.* Agona	infant milk products	37 case and 18 were hospitalized	France	[45]
*S.* Infantis	Raw chicken products	129 reported cases and 25 hospitalizations	32	[46]
*S.* Bovismorbificans	uncooked ham products	57 cases and 15 hospitalizations	Netherlands	[47]
*S.* Mbandaka	Kellogg’s Honey Smacks Cereal	135 reported cases and 34 hospitalizations	36	[48]
*S.* Enteritidis PT14b*	Egg and chicken products	287 reported cases and 78 hospitalizations	North West and South of England	[49]

*b: Information provided by the National Reference Centre for *Salmonella* (NRC), without confirmation that cause of death was attributable to *Salmonella* infection.

**Table 4:** Global Burden of salmonellosis.

*Salmonella* Serovars	Illnesses	Deaths	References
*S. enterica,* non-typhoidal	153,097,991	56,969	[72]
Invasive non-typhoidal *S. enterica*	596,824	63,312	[72]
Invasive non-typhoidal *S. enterica*	535,000	77,500	[71]
*S. enterica* Paratyphi A	4,826,477	33,325	[73]
*S. enterica* Typhi	20,984,683	144,890	[73]

**Table 5:** Cost of illness studies on salmonellosis.

Country	Year (S)	Cost	Reference
UK	2018	£0.21 billion	[78]
Sweden	2018	€25.6 million	[79]
Australia	2015	AUD 146.8 million	[80]
Canada	2000–2015	CAD 287.78 million	[81]
Netherlands	2012	€6.8 million	[82]
USA	2011	USD 394 million	[41]

**Table 6:** Legislations and Policies against Salmonellosis.

Organization	Regulations/Policies	Objective
European Commission	Regulation (EC) No 1177/2006	Overall implement acts on application of antimicrobial agents and vaccines for poultry birds
	Regulation (EC) No 2008/798/EC	Overall implement acts for importing live birds and eggs
	Regulation (EC) No 517/2011	Reduction in flocks of laying hens
	Regulation (EC) No 200/2010	Standard sampling and monitoring of *Gallus* *gallus* to reduce *Salmonella* among breeding stocks
	Decision (EC) No 1237/2007	Strict requirement mandating all eggs meant for trade must follow national control programs across the chain
	Regulation (EC) No 200/2012	Standard sampling and monitoring for reduction of *Salmonella* in broilers
	Regulation (EC) No 1190/2012	Standard sampling and monitoring for reduction of *Salmonella* in fattening and breeding turkeys
World Health Organization	Global Foodborne Infections Network (GFN)	Ensuring efficient oversight of antimicrobial-resistant *Salmonella* strains across the food chain; acquiring and testing samples along with data analysis
	WHO Advisory Group on Integrated Surveillance of Antimicrobial Resistance (AGISAR)	Working with FAO in prompt detection and response to food outbreaks by supporting national competent authorities at such periods
	International Network of Food Safety Authorities (INFOSAN)	Provides risk assessment data that serve as guidelines for international standards and recommendations through the Codex Alimentarius Commission

**Table 7:** Control measures recommended by the WHO.

Recommendations	Objectives
Prevention methods	Prevention steps should be applied at all stages of the food chain: from primary production, processing, distribution, sales and consumption.
	*Salmonella* prevention steps recommended in the food handlers handbook should be followed.
	The contact between children and domesticated animals require supervision.
	The public is advised to follow national and regional surveillance systems on foodborne diseases to be aware, detect and respond rapidly to salmonellosis outbreaks early and halt the spread.
Recommendations for the public and travellers	Food must always be cooked properly and served hot
	Only pasteurized milk and its products should be consumed
	Fruits and vegetables should be washed adequately before consumption
	Hands should be washed adequately after contacting animals or using the restroom.
	Ice meant for consumption must be made from potable water
Recommendations for food handlers	Food handlers should observe ingredients and follow hygienic food preparation rules.
	Provision of Five keys to safer food which provides a basis for food safety training courses both for professionals and consumers. They centre on: keeping clean, separating raw from cooked foods, cooking adequately, storing at correct temperatures and use of potable water
Recommendations for producers of fruits and vegetables	Practice good personal hygiene.
	Faecal pollution should be avoided
	Only treated faecal waste is permitted
	Irrigation water should be treated and well managed.
Recommendations for producers of aquaculture products	Practice good personal hygiene.
	Pond environment should be clean
	Water quality should be managed.
	Harvest equipment should be hygienic
	Ensure fish is healthy.

**Table 8:** Report on food handling practices.

Region	Study Type	Issues	References
South Africa (Hospital)	Interview using questionnaire	29% of all food handlers never had a food safety training course. More than 60% of the hospital staff had either good or satisfactory Food Safety Knowledge (FSK) but these did not contribute to better Food Safety Outcomes.	[99]
South Africa (Hospices)	Semi-structured questionnaire	68% had not taken basic food safety training. There was no knowledge of appropriate temperatures for refrigeration and hot RTE foods.	[100]
Ireland (Public)	Survey	Knowledge of food handling was below 10.8% and food poisoning below 20.1%—both were critically low.	[101]
Ethiopia	Survey	Unsatisfactory meet handling practice especially after smoking, sneezing, and coughing.	[102]
Norway, Denmark, Germany, Spain and the UK	Microbiological testing and Hygiene Performance Rating audits	Hygiene is a major issue in Slaughter Operational issues	[103]
Pakistan	Cross-tabulations, chi-square, and correlation tests.	Unhygienic vending practices for ready-to-eat foods	[104]
Global	Analysis of 81 full-text articles	Internalisation of food products across several countries increases risks for poor handling and food safety	[105]

**Table 9:** A comparison of food safety training efficacies.

Country	Training Method	Study Type	Behaviour	Conclusion	Reference
USA	Knowledge and behaviour-based online training video	Seven question quiz from Servy Safe coursebook	Observation by researcher	Behaviour-based training improves handwashing better than knowledge-based training especially during peak hours	[91]
Malaysia	Food safety training course based on regulations and behaviour training	31 questions	Self-reported questionnaire and researcher observations	Behaviour-based training performed better in certain areas than the control group	[112]
USA	Two hours ServSave training	Questionnaire	Self-reported	Volunteers reported a significant increase in food safety knowledge, but behaviour is unchanged. Self-reported data is unreliable	[36]
USA	Customized lessons using ServSafe	Questionnaire	Researcher Observation	Significant improvement in Food safety knowledge	[113]
Korea	Lecture and demonstrations	Questionnaire	Self-reported questionnaire and researcher observations	Increase in knowledge was statistically significant Intervention did not produce a change in behaviour	[64]
USA	Four hours ServSafe class and behaviour training	Questionnaire	Researcher Observation	Hand washing knowledge and behaviour significantly Improved but these did not improve general compliance behaviour	[114]

## Data Availability

Data sharing not applicable.
